# Serological Evidence of *Anaplasma phagocytophilum* and Spotted Fever Group *Rickettsia* spp. Exposure in Horses from Central Italy

**DOI:** 10.3390/pathogens8030088

**Published:** 2019-06-26

**Authors:** Valentina Virginia Ebani

**Affiliations:** 1 Department of Veterinary Science, University of Pisa, viale delle Piagge 2, 56124 Pisa, Italy; valentina.virginia.ebani@unipi.it; 2 CIRSEC, Center for Climatic Change Impact, University of Pisa, via del Borghetto 80, 56124 Pisa, Italy

**Keywords:** horses, *Anaplasma phagocytophilum*, spotted fever group *Rickettsia* spp., zoonosis, tick-borne infections

## Abstract

*Anaplasma phagocytophilum* and *Rickettsia* spp. are tick-borne bacteria of veterinary and human concern. In view of the One-Health concept, the present study wanted to evaluate the spreading of these pathogens in horses living in central Italy. In particular, the aim of the investigation was to verify the exposure to *A. phagocytophilum* in order to update the prevalence of this pathogen in the equine population from this area, and to spotted fever group (SFG) *Rickettsia* spp. to evaluate a possible role of horses in the epidemiology of rickettsiosis. Indirect immunofluorescent assay was carried out to detect antibodies against *A. phagocytophilum* and SFG (spotted fever group) *Rickettsia* spp. in blood serum samples collected from 479 grazing horses living in central Italy during the period from 2013 to 2018. One hundred and nine (22.75%) horses were positive for *A. phagocytophilum*, 72 (15.03%) for SFG *Rickettsia* spp., and 19 (3.96%) for both antigens. The obtained results confirm the occurrence of *A. phagocytophilum* in equine populations, and also suggest the involvement of horses in the epidemiology of SFG rickettsiosis. In both cases, in view of the zoonotic aspect of these pathogens and the frequent contact between horses and humans, the monitoring of equine populations could be useful for indication about the spreading of the tick-borne pathogens in a certain geographic area.

## 1. Introduction

Hematophagous arthropods, especially ticks, are well-known as vectors of several bacterial, viral, and protozoan pathogens. Among them, *Anaplasma phagocytophilum* and *Rickettsia* spp. may induce clinical manifestations in humans and different animal species.

*Anaplasma phagocytophilum* is an obligate intracellular, Gram negative bacterium belonging to the order *Rickettsiales*, family *Anaplasmataceae*. It is able to infect granulocytes, mainly neutrophils, of several domestic and wild animal species [1]. 

Wild mammals usually serve as asymptomatic reservoirs of *A. phagocytophilum*, whereas domestic animals may develop clinically defined diseases, such as tick-borne fever in cattle and sheep and granulocytic anaplasmosis in dogs [2]. Furthermore, *A. phagocytophilum* is cause of infection in human beings, who develop a disease called human granulocytic anaplasmosis (HGA), varying from mild to severe forms with fever, headache, myalgia, arthralgia, leukopenia, and thrombocytopenia; moreover, serious opportunistic infections can occur in immunocompromised patients during the course of HGA [3].

Horses infected by this pathogen develop a disease known as equine granulocytic anaplasmosis (EGA) (formerly equine granulocytic ehrlichiosis), characterized by a wide range of clinical signs. Usually, infected horses show fever, lethargy, ataxia, reluctance to move, icterus, and petechiation; laboratory blood abnormalities may include leukopenia, thrombocytopenia, and anemia [4].

In all animal species, *A. phagocytophilum* is transmitted by a tick bite. *Ixodes* species are involved in the epidemiology of this pathogen worldwide, in particular *I. ricinus* in Europe, including Italy [2].

The genus *Rickettsia* comprises obligate intracellular, Gram-negative bacteria transmitted by hematophagous arthropods. Spotted fever group (SFG) includes several *Rickettsia* species responsible for disease, often serious, in animals and humans. *Rickettsia conorii* is the etiologic agent of the Mediterranean spotted fever (MSF) that represents the most widespread SFG rickettiosis in the Mediterranean countries, including Italy, especially in the southern (Sardinia, Sicily, Calabria) and central regions [5], where most cases have been reported mainly between the months of June and September [6].

In Italy, several SFG rickettsiae are circulating, as mainly demonstrated by molecular investigations on tick populations. In particular, DNA of *R. conorii, R. helvetica*, *R. massiliae*, *R. slovaca*, *R. monacensis*, *R. aeschlimannii*, *R. raoultii*, and *R. africae* have been detected [7,8,9,10,11,12].

No data about the spreading of rickettsiae among horses living in Italy is available, and very scant information about equine rickettsiosis in other countries is present in the scientific literature [13,14].

Considering that anaplasmosis and rickettsiosis are zoonotic infections and the high occasion of contact between humans and horses, in view of the One-Health concept, the present study wanted to evaluate the spreading of these pathogens in horses living in central Italy. In particular, the aim of the investigation was to verify the exposure to *A. phagocytophilum* in order to update the prevalence of this pathogen in equine population from this area, and to SFG *Rickettsia* spp., to evaluate a possible role of horses in the epidemiology of rickettsiosis.

## 2. Results

Among the 479 tested horses, 109 resulted positive for *A. phagocytophilum*, with 22.75% total mean seroprevalence; prevalence values observed in the different years varied from 17.46% (2013) to 27.08% (2018). Antibody titers ranged from 1:40 to 1:1280. 

Seventy two (15.03%) horses had antibodies to SFG *Rickettsia* spp. Prevalence values ranged from 11.26% (2016) to 17.71% (2018) in relation with the years of sampling. Furthermore, antibody titers from 1:64 to 1:1024 were observed. Nineteen (3.96%) horses had antibodies to both *A. phagocytophilum* and SFG *Rickettsia* spp. Results are summarized in Table 1, Table 2 and Table 3.

## 3. Discussion

Tick-borne diseases are major animal and human health issues in several geographic areas. Global warming has deeply influenced the spread of hematophagous arthropods, including ticks, but other factors are involved in their distribution. Animals’ movements, agricultural and wildlife management, and urbanization with consequent reduction of natural areas have determined changes in tick distribution, with increasing presence in urban and peri-urban environment.

Horses examined in this study lived in areas with environmental conditions which favor tick diffusion, especially abundant vegetation and presence of other animal species, mainly wildlife. Several tick species are present in this area, including *Ixodes ricinus*, which is the main vector of *A. phaocytophilum* [2] and is also involved in the transmission of rickettiae. Furthermore, the brown dog tick *Rhipicephalus sanguineus*, the main vector of *R. conori*, may be found not only among dogs, but also in wildlife [15].

Results obtained during this survey showed that the examined horses had been exposed to the investigated tick-borne pathogens, with a higher seroprevalence detected for *A. phagocytophilum* than for SFG *Rickettsia* spp. Higher values of seroprevalence for both pathogens were detected among samples collected in 2017 and 2018. These results could be related to climatic conditions that allowed a higher presence of ticks in the areas where the tested horses lived. However, the spread of arthropods is related to further factors (presence of other animals, environmental management, acaricide treatments) that were not fully known in this study.

Previous investigations found *A. phagocytophilum* infection in equine populations in Italy: in detail, serological surveys detected prevalence values ranging between 9% and 17%, whereas molecular studies found prevalences from 4.7% to 25.62% [16,17,18,19,20]. Seroprevalences found in Europe, which ranged from 11.3% to 20%, were quite similar [21,22,23]; conversely, molecular surveys carried out in some European countries detected lower prevalences that varied from 1.4% to 9.8% [24,25,26].

Furthermore, *A. phagocytophilum* infection was detected in other animal species living in Italy [27,28,29,30], as well as *A. phagocytophilum* DNA being found in ticks collected from animals or environment [31]. The present results confirm the spreading of this tick-borne pathogen among equine populations in the investigated areas. Moreover, considering that the tested animals did not show clinical signs, the results corroborated that asymptomatic forms may be developed by infected horses. In fact, some authors affirm that horses from endemic areas have a higher seroprevalence to *A. phagocytophilum* than those from non-endemic areas, and horses introduced into an endemic area are more likely to develop illness than native horses [32]. However, clinical findings, when present, are not specific, and it can be difficult to differentiate EGA from other diseases, mainly piroplasmosis. Moreover, horses infected by *A. phagocytophilum* are predisposed to developing secondary infections, which may complicate the clinical diagnosis [32].

Data about the presence of SFG *Rickettsia* spp. in Italy mainly concern humans [33]. Some studies have been carried out in ticks and wild animals, and case reports of canine rickettsiosis have also been documented [34,35]. Data about rickettiosis in Italian equine populations are not available in the scientific literature, and very scant studies have been reported from other European countries. Elfving et al. [13] tested sera from 63 horses in Sweden with indirect immunofluorescence assay (IFA) employing *R. helvetica* as antigen, and found a 36.5% prevalence; Skotarczack et al. [14] found *R. helvetica* and *R. monacensis* DNA in two ticks collected from ponies in Poland.

Furthermore, serological surveys have been carried out in horses living in South America. A 2.85% seroprevalence for SFG *Rickettsia* spp. was found in horses from Colombian Orinoquia [36]; in Brazil, 183/504 (36.3%) horses were seropositive for *Rickettsia rickettsii* [37], whereas among 258 horses tested for *R. rickettsii*, *R. amblyommatis*, and R. *bellii*, 152 (58.91%) were seroreactive for at least one *Rickettsia* species [38].

To the best of our knowledge, this is the first serological survey on the occurrence of SFG *Rickettsia* spp. in horses from Italy. Finding *Rickettsia*-positive horses suggests that they can contribute to the natural cycle of these bacteria as hosts for infected ticks. Equine illness related to rickettsia has never been described, therefore horses could respond immunologically to exposure to rickettsiae without developing clinical signs.

An experimental infection of horses with *R. rickettsii* demonstrated that the animals had no bacteremia, clinical, hematological, or blood biochemical alterations, but they had specific antibodies from 10 days to 2 years after infection [39].

During the present investigation, *R. conorii*, which belongs to the SFG, was used as an antigen for IFA. This pathogen, an agent of MSF disease, has been frequently found in Italy, as demonstrated by serological and molecular studies [33,35,40,41].

Horses resulting positive for rickettsiosis could have antibodies to *R. conorii*, as well as to other SFG *Rickettsia* spp. present in Italy, because IFA, even though considered the gold standard method for the serological diagnosis of rickettsiosis [42], is not able to differentiate between antibodies against the different SFG species [5].

Horses are largely employed in agonistic activity and are frequently maintained for recreational purposes, therefore, humans are highly exposed to the risk of being bitten by ticks previously fed on infected horses, as suggested by some authors who have considered human contact with horses as a risk factor for acquiring tick-borne lymphadenopathy (TIBOLA) caused by *R. slovaca* [43].

Detection of horses with antibodies to both *A. phagocytophilum* and SFG *Rickettsia* spp. confirms that the equine population, as well as human beings and other animals, may be affected by more arthropod-borne pathogens as consequence of the transmission by one tick harboring different microorganisms and/or more infected ticks. *A. phagocytophilum*, *Borrelia burgdorferi* sensu lato, *Coxiella burnetii*, and piroplasms have been demonstrated to be responsible for co-infections in clinically healthy and symptomatic horses [19,20,22]. Further studies could be useful to investigate if rickettsiae may complicate equine clinical forms when involved in co-infections.

## 4. Material and Methods

### 4.1. Animals

From January 2013 to December 2018, peripheral whole blood samples were collected from 479 grazing horses. Animals were actively racing and lived in various farms and horse centres located in lowland and hilly areas of Central Italy; they did not show clinical signs and were not under antibiotic treatment. Breeders and owners reported previous tick exposure.

Whole blood samples (about 10 mL), drawn from the right or left jugular vein, were centrifuged at 1500× *g* for 15 min, and the sera were collected and immediately tested or stored at −20 °C. 

#### Ethical Statement

The collection of blood samples was executed for other clinical exams as part of routine health care by collaborating veterinarians during clinical visits. All animals were treated with standard practices of animal care and no horses were submitted to blood collection for this study only. However, in all cases, informed consent was obtained from the owners.

### 4.2. Serological Analyses

The indirect immunofluorescence antibody test (IFA) was executed on IFA slides prepared with *Anaplasma phagocytophilum* and *Rickettsia conorii* (Fuller Laboratories Fullerton, California, USA) antigens, respectively.

Blood sera were diluted 1:40 and 1:64 in phosphate-buffered saline (PBS, pH 7.2), considered cut-off values for *A. phagocytophilum* and *Rickettsia* spp., respectively, as reported in previous studies [13,44]. The test was executed employing a rabbit fluorescein isothiocyanate-conjugated anti-horse IgG (Sigma-Aldrich, Milano, Italy) diluted 1:30 in Evans Blue (Sigma-Aldrich) solution and following the manufacturer’s procedure. Samples scored positive were two-fold serially diluted to determine the endpoint titre. 

### 4.3. Statistical Analysis 

Statistical evaluation was carried out by the χ^2^ test to analyze the results of serological tests in relationship to the years in which samples were collected. Values of *P* < 0.05 were considered significant.

## 5. Conclusions

Grazing horses living in central Italy seem to be frequently exposed to ticks, and consequently to pathogens transmitted by these hematophagous vectors. The present results confirm the spreading of *A. phagocytophilum* in the investigated geographic area, where the bacterium has been previously found in various animal populations.

Moreover, horses scored positive to SFG *Rickettsia* spp. suggest that they can be infected by this microorganism, too, even though they do not develop disease. 

In all cases, infected horses may be involved in the epidemiological cycle of *A. phagcoytophilum* and SFG species, as *R. conorii* and other rickettsiae that were considered non-pathogenic for decades and now are associated with human infections. 

Considering that the spreading of tick-borne diseases is a growing concern, and in light of the One-Health concept, it is necessary to increase the surveillance of these infections in animals, not only pets but also horses, that have frequent contact with humans.

## Figures and Tables

**Table 1:** Results obtained by indirect immunofluorescence test for *Anaplasma phagocytophilum* in relation to years of sampling and antibody titers.

Years	N. Tested Horses	N. Positive (%) Horses	Antibody Titers (%)					
			1:40	1:80	1:160	1:320	1:640	1:1280
2013	63	11 (17.46)	2	7	1	1	-	-
2014	76	15 (19.73)	3	8	3	-	1	-
2015	92	22 (23.91)	5	13	2	1	-	1
2016	71	14 (19.71)	3	8	1	2	-	-
2017	81	21 (25.92)	4	15	1	-	-	1
2018	96	26 (27.08)	7	11	5	1	2	-
Total	479	109 (22.75)	24 (5.01)	62 (12.94)	13 (2.71)	5 (1.04)	3 (0.63)	2 (0.42)

**Table 2:** Results obtained by indirect immunofluorescence test for spotted fever group (SFG) *Rickettsia* spp. in relation to years of sampling and antibody titers.

Years	N. Tested Horses	N. Positive (%) Horses	Antibody Titers (%)				
			1:64	1:128	1:256	1:512	1:1024
2013	63	9 (14.28)	5	3	-	1	-
2014	76	13 (17.10)	4	5	3	-	1
2015	92	11 (11.95)	6	5	-	-	-
2016	71	8 (11.26)	3	3	2	-	-
2017	81	14 (17.28)	4	2	5	3	
2018	96	17 (17.71)	8	6	2	-	1
Total	479	72 (15.03%)	30 (6.26)	24 (5.01)	12 (2.50)	4 (0.84)	2 (0.42)

**Table 3:** Horses resulted positive to both *Anaplasma phagocytophilum* and SFG *Rickettsia* spp. with indirect immunofluorescence test.

Horses	*Anaplasma phagocytophilum*	SFG *Rickettsia* spp.
1	1:80	1:64
2	1:80	1:64
3	1:80	1:128
4	1:40	1:64
5	1:160	1:64
6	1:40	1:256
7	1:80	1:128
8	1:160	1:128
9	1:80	1:128
10	1:80	1:64
11	1:80	1:64
12	1:80	1:128
13	1:40	1:128
14	1:40	1:64
15	1:320	1:64
16	1:40	1:64
17	1:40	1:256
18	1:80	1:128
19	1:80	1:64
